# Progress in Medicine

**Published:** 1897-04-24

**Authors:** 


### Progress in Medicine.

### NEUROLOGY.

**Locomotor Ataxy.**—It has long been known that if optic atrophy occurs as an early symptom in locomotor ataxy the spinal malady often becomes stationary. Bailey[1] confirms this from an extended investigation, in which he comes to the following conclusions. In about 75 per cent. of cases of tabes in which optic atrophy is an early symptom, some of the other symptoms, especially inco-ordination of movement, may be late in appearing or may not develop at all; but it may develop simultaneously with or immediately succeed the blindness. He further finds that the association with the optic atrophy of oculo-motor palsies is of no prognostic significance.

Some three years ago Hitzig first described anæsthesia of the trunk as a fairly constant symptom in locomotor ataxy, and the subject has recently been investigated again by Patrick.[2] The sensory blunting of the extremities in tabes has long been known, and is quite distinct from this trunk anæsthesia, which occurs as a band about the body, generally in the region of the nipple, in about 85 per cent. of cases of tabes. The anæsthesia does not correspond to the cutaneous distribution of the intercostal nerves, but to the innervation of the skin by spinal cord segments, agreeing with the zones marked out by cases of herpes, and by the referred pain and tenderness of visceral disease. In a few of the cases a narrow band of hyperæsthesia may exist on either side of the anæsthetic region.

**Disseminated Sclerosis.**—Buzzard[3] has recently again called attention to the great liability of mistaking a case of disseminated sclerosis in an early stage for one of hysteria, and makes some most valuable remarks on the distinguishing characters. In an early stage of disseminated sclerosis intermissions may occur, even for long periods; thus there may be a temporary paresis of one limb, subsequently followed, perhaps, by paresis of another. Intention tremor is a most characteristic feature of disseminated sclerosis, for though in hysteria there may be a clumsiness of movement, due to loss of muscular sense, it very rarely assumes the character of a rhythmic tremor. Nystagmus is almost conclusive evidence of organic disease in any case of supposed hysteria, though of course it occurs not merely in disseminated sclerosis, but also in diseases of the cerebellum and corpora quadrigemina, Friedrich's ataxy and chronic alcoholism, and also in miners. Again, ankle clonus, if strongly marked, is conclusive of organic disease, though, if imperfect, it may be of functional origin. Of greater weight than any of these symptoms is atrophy, even though slight, of the optic disks, which is usually associated with either a central scotoma or concentric narrowing of the visual field.

Cassirir,[4] of Oppenheim's clinic, draws attention to the occasional difficulty of distinguishing between disseminated sclerosis and cerebro-spinal syphilis. Ocular symptoms and spastic paralysis, for instance, may occur in both affections. The distinguishing points are the intention tremor, nystagmus and scanning speech of disseminated sclerosis, and the frequent remissions in syphilis—though, as Buzzard has shown, this last phenomenon may and often does occur in disseminated sclerosis. But where these characteristic features of disseminated sclerosis are absent, as they may be, a differential diagnosis may be impossible, at least, for some time. Even in these cases the lateness of onset and the history may point, though not definitely, to cerebro-spinal syphilis.

The pathology of disseminated sclerosis has recently been investigated by Redlich,[5] who says that the sclerotic patches exhibit many variations, which may be broadly divided into three classes, the first of which is characterised by the appearance of many fine parallel fibres, among which a few uninjured nerve fibres and axis cylinders are to be seen. In the second class the patches are formed by the thickening of normal glia structure, and the blood-vessels and capillaries are frequently thickened in the neighbourhood. In the third class there is a large-meshed glia tissue with empty meshes, caused apparently by relatively speedy destruction of the axis cylinders. This last class is apparently different from the other two from a pathological point of view.

The etiology of disseminated sclerosis is still very obscure and quite undetermined. On the one hand, Oppenheim[6] lays stress on the importance of various intoxications as a cause. Thus of 28 cases, 11 had been exposed to the influences of such poisons as lead, zinc, copper, &c. He also thinks it may follow infectious diseases, such as influenza, or be the result of trauma. On the other hand, Strümpell,[7] whilst admitting its occasional occurrence after specific fevers, does not think these are the usual causes, nor does he think that a toxic origin or a primary affection of the blood vessels is probable. He is of opinion that it is of congenital origin—endogenous, not exogenous. He has been led to this conclusion by the fact that it commences as a rule early in life, and by a case in which it was associated with hydromyelia, and lastly because, whereas exogenous diseases affect the axis cylinders or nerve cells very early, these structures are spared until very late in disseminated sclerosis. The conclusion come to, then, is that it starts in the neuroglia as a multiple gliomatosis of congenital origin. Evidently acute illnesses, trauma, &c., are not excluded by this theory from the etiology, for they may act as exciting causes.

**Syringomyelia.**—Dercum and Spiller[8] record a most interesting case of syringomyelia with asthropathy. The patient, who died in 1895 at the age of 58 years, began to suffer from pains and weakness in his legs in 1883, some three years after straining his back whilst lifting some heavy weights. He gradually developed spastic paraplegia, with rigidity and weakness in his legs, with impairment of sensation and loss of control over the sphincters. In 1888 his right shoulder became swollen, and there was some grating and effusion, but no pain. The effusion slowly increased, and finally the capsule ruptured, leading to a subglenoid dislocation and effusion into the surrounding tissues. He finally died of exhaustion in 1895. On examination of the spinal cord, the authors found gliosis round an enlarged central canal, extending from the lower end to the first thoracic segment; above that level the cavity was limited to the right posterior horn. There was also considerable thickening of the membranes in the lumbar regions, and degeneration of the pyramidal tracts and columns of Goll. In the right shoulder joint the head and surgical neck of the humerus had entirely disappeared, and there was enlargement, with lipping of the edges, of the glenoid fossa.

**Lead Poisoning.**—Abram[9] gives a short account of three cases of lead poisoning, and a note on a simple method of detecting lead in the urine. The first two patients were examples of the cerebral form of lead poisoning; the first of the epileptiform, and the second of the comatose variety, and all three cases hardly call for further remark. But the method of testing for lead is so simple and accurate that it is worthy of notice. It is adapted by Abram from Von Jaksch. A strip of pure magnesium is placed in the urine or other organic fluid. Ammonium oxalate is added in the proportion of 1 gramme to each 150 cubic centimetres of the fluid. If lead is present it is deposited on the magnesium, and can be seen within an hour. Confirmatory tests are to warm the slip with a crystal of iodine, when a yellow iodide of lead is formed, or dissolve in nitric acid and apply the usual tests.

Da Costa[10] gives a most instructive account of a case of rapidly-occurring hemiplegia from acute lead poisoning. The patient was a lady thirty-five years old, in very good health. Some rooms in her house were painted, and the house became filled with the smell. She quickly began to suffer from headache, and in the course of three days a right hemiplegia developed—tongue, arm, and leg. This had been preceded by numbness. Shortly afterwards there was a sense of numbness and coldness in the right leg, but no motor weakness developed. Four weeks subsequently the tendon jerks of the right arm and leg were exaggerated. and there was paresis of the arm and leg, especially of the latter. There was no anæsthesia, nor were there any tender spots in the course of the nerves, There was a bluish line round the gums. Under iodides and sulphate of magnesium gradual recovery took place. The shortness of exposure before symptoms developed and the form of paralysis—hemiplegia—are exceptional, as is also the affection of the central nervous system without preceding colic. The diagnosis of these cases has to be made, both from those cases of peripheral neuritis due to lead, and from those cases of chronic lead poisoning in which as a consequence of renal cirrhosis cerebral hæmorrhage results. The condition is easily differentiated from peripheral neuritis by its hemiplegic distribution, by the absence of degeneration of the muscles, and by the exaggerated tendon jerks; and from the latter condition by the absence of the signs of chronic renal disease, and by the subacute—not sudden—onset.

**Tumours of the Brain.**—Williamson[11] has recorded three cases of tumour of the prefrontal region of the brain, and has given a most valuable digest of the symptomology of tumours of that region derived from an analysis of fifty cases. Headache was marked in most cases, and was generally frontal, but sometimes occipital. Optic neuritis was almost invariably present and it was unilateral or much more advanced on one side than the other in the great majority of cases. This inequality in the affection of the disks is of great importance in diagnosis. Unilateral or bilateral loss of smell was noticed in seven only, whilst unilateral localised swelling in the frontal region and unilateral exophthalmos were still more rare. Motor symptoms were slight when present, and only occurred in some cases as a result of extension backwards of the tumour into the motor region. Anæsthesia was never present. Ataxia was sometimes present of a type similar to that produced by cerebellar lesions, and a further point of similarity to cerebellar tumours was the variability in the knee jerks, which were absent in some cases (20 %), in some normal, and in others increased. Psychical symptoms were generally well marked and often occurred early. In some cases there was childish behaviour with an abnormal tendency to fall asleep, in others mental impairment with a peculiar hilarity and tendency to jest. Diagnosis from cerebellar tumour may be difficult, for prefrontal and cerebellar tumours may have in common occipital headache, present knee jerks, and ataxy. The points of distinction are the early and predominant mental impairment, and the greater affection of the disk on one side than on the other, and the rarity of paresis of the limbs and of convulsions in prefrontal lesions. From lesions of the motor area the differential points are the earlier onset of mental symptoms, the neuritis, and the occasional loss of knee jerks. An operation was performed in three out of the fifty cases, two of which were successful, one being an abscess, and the other a solid tumour.

[1] Bailey, Med. Record, Nov. 14, 1896. [2] Patrick, New York Med. Jour., Feb. 6, 1896. [3] Buzzard, Lancet, Jan. 2, 1897. [4] Cassirir, Deut. Med. Wochenschrift, Oct. 22, 1896. [5] Redlich, Neur. Centralblatt, p. 562, 1896. [6] Oppenheim, Berlin Klin. Wochenschrift. [7] Strümpell, Neurol, Centralblatt, Nov., 1896. [8] Dercum and Spiller, Amer. Jour. Med. Scien., Dec., 1896. [9] Abram, Lancet, Jan. 16, 1897. [10] Da Costa, Amer. Jour. Med. Scien., Feb., 1897. [11] Williamson, Brain, Autumn, 1896.

